# First clinical insights into pulsed field ablation for pulmonary vein stumps

**DOI:** 10.1002/joa3.70067

**Published:** 2025-04-11

**Authors:** Keigo Misonou, Masato Fukunaga, Jun Hirokami, Kenichi Hiroshima, Kenji Ando

**Affiliations:** ^1^ Department of Cardiology Kokura Memorial Hospital Kitakyusyu Fukuoka Japan

**Keywords:** atrial fibrillation, intracardiac echocardiography, lung lobectomy, pulmonary vein stump, pulsed field ablation

## Abstract

It has been reported that pulmonary vein stumps after lung lobectomy can act as triggers for atrial fibrillation. These two cases represent the first report of successful pulmonary vein isolation by pulsed field ablation using the pentaspline catheter after pulmonary lobectomy under intracardiac echocardiography guidance.

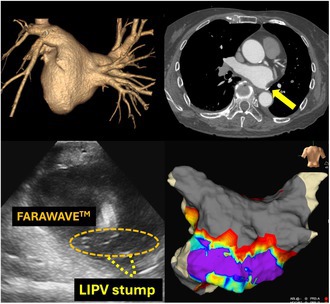

Residual pulmonary vein (PV) stumps following pneumonectomy remain electrically active and can be triggers for atrial fibrillation(AF). However, pulmonary vein isolation (PVI) in patients with severed PVs remains challenging for AF ablation. Effective radiofrequency PVI in a series of 15 cases^1^ and cryoballoon ablation in one case have recently been reported,^2^ though none with pulsed field ablation (PFA). This report provides clinical insights into the PFA using the FARAPULSE™ PFA System (Boston Scientific) for PV stump isolation. Here, we present two cases of paroxysmal AF following lower pulmonary lobectomy, in which PVI and left atrial posterior wall isolation (PWI) were performed using the pentaspline catheter under intracardiac echocardiography (ICE) guidance.

A 72‐year‐old female underwent a left lower lobectomy for lung cancer 12 years ago. On transthoracic echocardiography (TTE), the left atrial diameter (LAD) was 39 mm, and the left atrial volume (LAV) was 51 mL. Preoperative contrast‐enhanced computed tomography (CT) confirmed that the left inferior pulmonary vein was a PV stump (Figure 1). The procedure was performed under deep sedation. After femoral vein punctures, the left atrium (LA) was accessed through a single transseptal puncture with a transseptal needle under the ICE guidance. After a successful transseptal puncture, the transseptal sheath was exchanged for a 13‐F steerable sheath (FARADRIVE™). A 12‐F over‐the‐wire ablation catheter (FARAWAVE™) was then introduced in the LA guided by a J‐tip guidewire of 0.035‐inch diameter. Following PV cannulation with the wire, ablation was performed with four applications in the basket configuration and four applications in the flower configuration for each PV. For the PV stump, since wire guidance was challenging, applications were performed without wire cannulation. Subsequently, the 3D electroanatomical mapping (EAM) of the LA was created using the EnSite™ 3D‐mapping system (Abbot) with the Advisor™ HD Grid catheter (Abbott). The 3D‐EAM revealed that the posterior wall (PW) was nearly isolated and demonstrated an unexpected isthmus. Therefore, additional ablation was performed to achieve complete isolation of the PW by adding applications to the PW in a flower configuration under ICE guidance. The postablation EAM confirmed that the PW was completely isolated (Figure 2).

**FIGURE 1:**
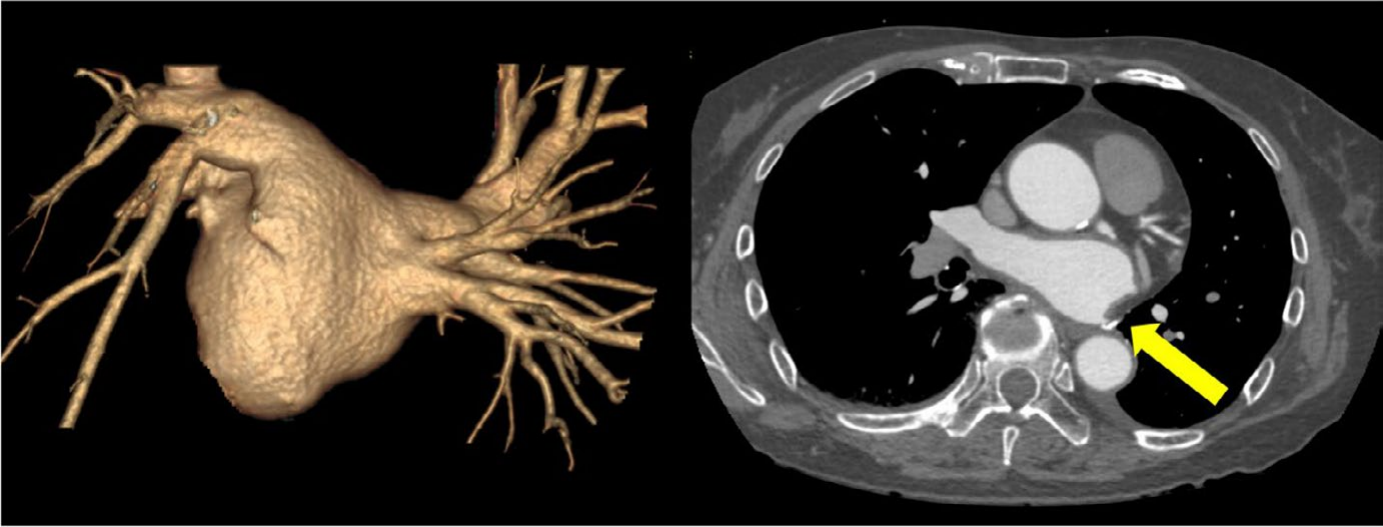
Case 1 had undergone left lower lobectomy, resulting in the left inferior PV being transected. Contrast‐enhanced CT shows that the left inferior PV was the PV stump (yellow arrow).

**FIGURE 2:**
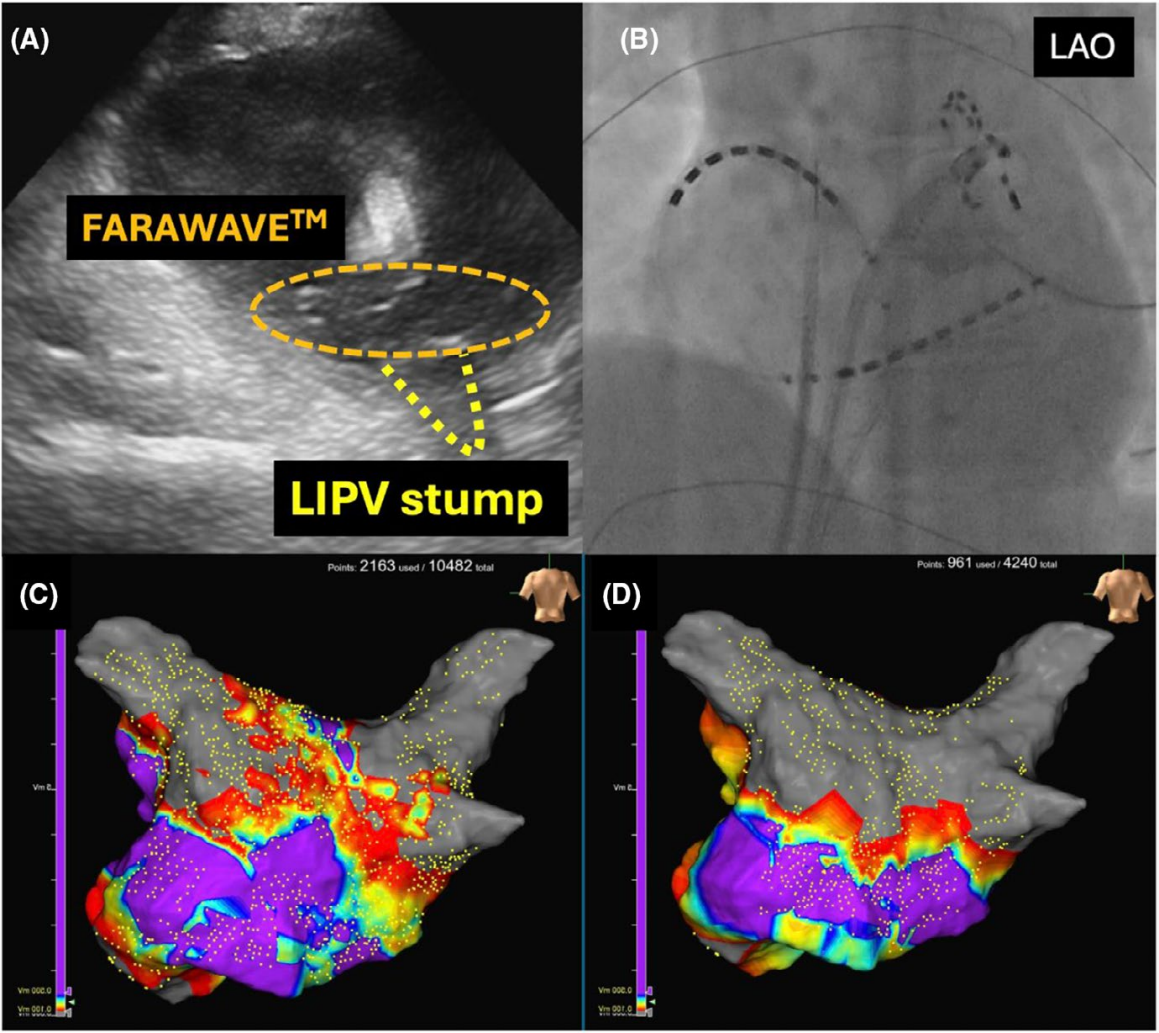
(A) Intracardiac echocardiography (ICE) confirmed that the pentaspline catheter (FARAWAVE™) was in contact with the left inferior pulmonary vein antrum. (B) The fluoroscopic image shows that the FARAWAVE™ catheter in a flower configuration is positioned at the LIPV in the LAO view. (C) The voltage map of the left atrium (LA) after the pulmonary vein isolation (PVI) obtained with Advisor™ HD Grid (PA view). The 3D‐EAM revealed that the left atrial posterior wall was nearly isolated and demonstrated an unexpected isthmus on the posterior wall. (D) The map after additional applications to the posterior wall showed that the left atrial posterior wall was completely isolated.

A 60‐year‐old male patient who had been diagnosed with paroxysmal AF 10 years ago. He underwent a right lower lobectomy for lung cancer 3 months ago. On TTE, the LAD was 31 mm, and the LAV was 56 mL. Pre‐procedural contrast‐enhanced CT confirmed that the right inferior PV was a PV stump (Figure 3). For the nontransected PVs, four applications were performed in the basket configuration and four applications in the flower configuration, while for the PV stumps, applications were performed in the basket configuration under ICE guidance. In this case, the wire could not be inserted into the PV, resulting in poor catheter maneuverability. As a result, the flower configuration did not fit properly, causing part of the spline to enter the PV stump. Therefore, the basket configuration was used, and since good contact was confirmed by ICE, the application was performed using the basket configuration. Subsequently, 3D‐EAM of the LA was created using the CARTO 3™ 3D‐mapping system (Biosense Webster) with the OCTARAY™ catheter (Biosense Webster). The post‐PVI map showed the low voltage zone in the inferior part of the PW. Therefore, additional ablation was performed to achieve bidirectional block of the inferior line in order to prevent a macro‐reentrant atrial tachycardia (AT) (Figure 4).

**FIGURE 3:**
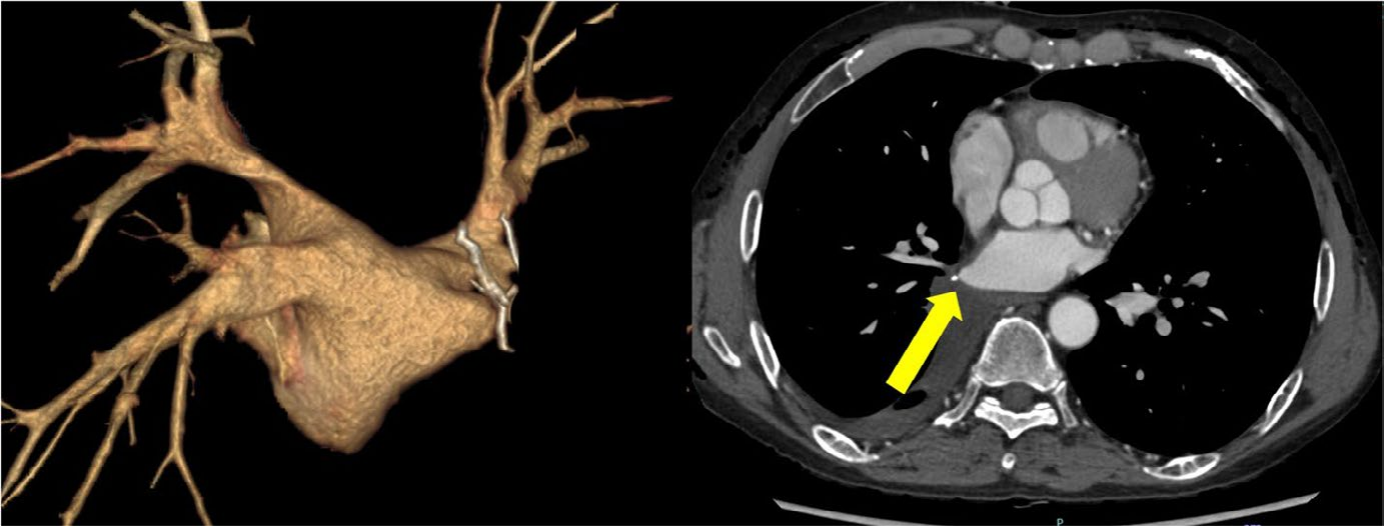
Case 2 had undergone right lower lobectomy, resulting in the right inferior pulmonary vein (PV) being transected. Contrast‐enhanced CT shows that the right inferior PV was the PV stump (yellow arrow).

**FIGURE 4:**
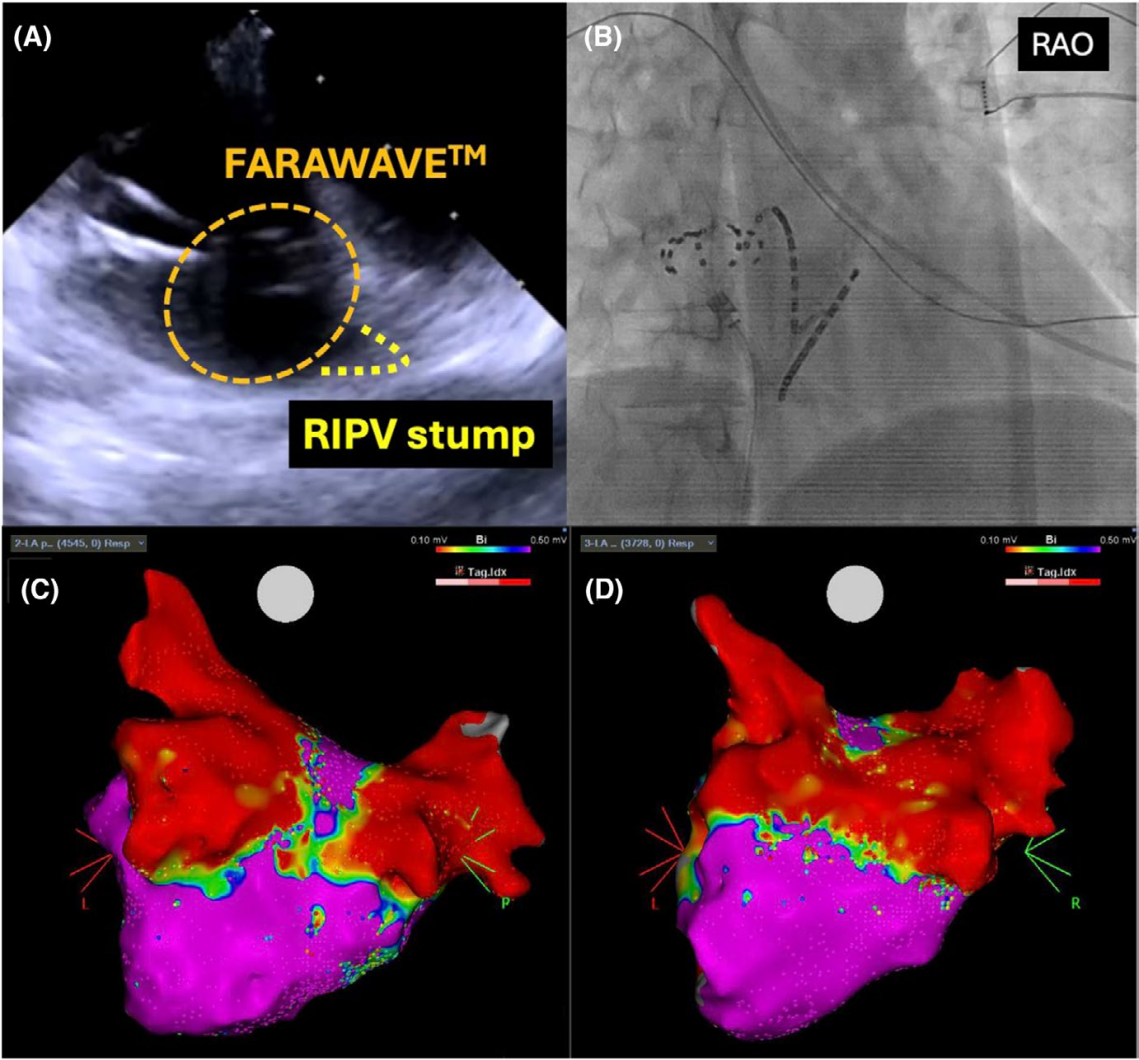
(A) Intracardiac echocardiography (ICE) confirmed that the pentaspline catheter (FARAWAVE™) was in contact with the right inferior pulmonary vein (PV) antrum. (B) The fluoroscopic image shows that the FARAWAVE™ catheter in a basket configuration is positioned at the RIPV in the RAO view. (C) Voltage map of the left atrium (LA) after the PVI obtained with OCTARAY™ (PA view). The 3D‐EAM revealed that the left atrial posterior wall was nearly isolated and demonstrated an unexpected isthmus on the posterior wall. (D) Additional applications rendered the inferior line of the left atrial posterior wall.

In patients with AF and previous lobectomy or pneumonectomy, identification and isolation of all PVs are challenging. In studies using radiofrequency ablation or balloon ablation, it has been reported that in 9 out of 19 patients (47.4%) with a history of pulmonary lobectomy or pneumonectomy, at least one PV remnant could not be identified, leading to incomplete isolation and reduced arrhythmia‐free survival.^3^ Unlike balloon ablation, PFA using the pentaspline catheter does not require confirmation of PV occlusion, making isolation of PV stumps easier and safer. Furthermore, stenosis or occlusion of any remaining PVs other than the PV stump may be a critical complication that occurs in thermal ablation. However, PFA does not raise concerns about PV stenosis. In our two cases, the anatomical location of the PV stump was identified by pre‐procedural CT. Intraoperatively, its position and contact with the catheter were confirmed using ICE, ensuring successful PVI with the pentaspline catheter.

After PVI, both cases showed that the PW was nearly isolated. In cases where the lower PVs have been transected, there is a possibility of unintentionally forming a substrate on the PW that contributes to iatrogenic slow conduction. We have considered that there are two main reasons for this. The first is that in patients who have undergone PV resection, the wire cannot be inserted into the PV, leading to insufficient catheter stability, which results in the formation of an unintentional broader lesion compared with typical cases. The second reason is that both cases in this study had small LA (LAD of 39 mm and 31 mm), which resulted in a relatively short distance between the left and right PVs. Tohoku S, et al. have reported that after PVI using PFA, macro‐reentrant AT associated with the PVI lesion set on PW was commonly observed, which implied that the extensive lesion set on PW might create an unexpected slow conduction zone leading to recurrent AT.^4^ Therefore, we added PWI with the pentaspline catheter to prevent the recurrence of AT. In postlobectomy patients, to assess the PW lesion, it may be beneficial to use 3D‐EAM. Recently, the pentaspline PFA catheter has been shown to be safe and effective for PVI and PWI in patients with persistent AF.^5^ Therefore, when a substrate of AT is formed on the PW, additional PWI using the pentaspline catheter should be considered.

PFA using the pentaspline catheter, guided by ICE and contrast‐enhanced CT, represents a viable and safe approach to isolating PV stumps in postlobectomy patients. These cases demonstrate its clinical feasibility and underscore its potential to address complex anatomical challenges. However, prospective studies are necessary to evaluate long‐term outcomes and further refine this technique.

## FUNDING INFORMATION

None.

## CONFLICT OF INTEREST STATEMENT

The authors declare no conflict of interest for this article.

## PATIENT CONSENT STATEMENT

The patient has provided consent for publication.

## Data Availability

The data supporting the findings of this study are available from the corresponding author upon reasonable request.
